# Xenograft Enriched with Autologous Bone Marrow in Inlay Reconstructions: A Tomographic and Histomorphometric Study in Rabbit Calvaria

**DOI:** 10.1155/2012/170520

**Published:** 2012-08-29

**Authors:** Marcelo de Oliveira e Silva, André Antonio Pelegrine, Alexandre Alves Pinheiro da Silva, Luiz Roberto Manhães Júnior, Rafael de Mello e Oliveira, Silvana Gaiba França, Antonio Carlos Aloise, Lydia Masako Ferreira

**Affiliations:** ^1^Medicine School, Unifesp, Rua Pedro de Toledo, 781-11 andar, 04039-032 São Paulo, SP, Brazil; ^2^Unesp Dental School, 04039-032 São José dos Campos, SP, Brazil

## Abstract

*Objective*. The aim of this study was to evaluate the bone healing after the usage of a scaffold enriched with bone marrow. *Study Design*. Ten rabbits were divided into 2 groups of 5 animals. Bilateral 12 mm diameter defects were created in the parietal bones. In control group Bio-Oss were inserted in both defects and, in experimental group, Bio-Oss enriched with autologous bone marrow were inserted in both defects. In these two groups, one of the calvarial defects was covered with Bio-Gide. The rabbits were sacrified 8 weeks after surgery and both CT and histomorphometric analysis were done. *Results*. The CT showed a lower remaining defect area in the experimental group covered with Bio-Gide when compared with control group, with and without Bio-Gide. The histomorphometrics showed no difference between groups regarding the non-vital mineralized tissue area. For vital mineralized tissue area, the experimental group covered with Bio-Gide obtained a higher percentage area when compared with control group, with and without Bio-Gide. For non-mineralized tissue area, the experimental group covered with Bio-Gide obtained a lower percentage area when compared with control group, with and without Bio-Gide. *Conclusion*. Both autologous bone marrow and membrane can contribute to the enhancement of bone healing.

## 1. Introduction

Bone defects are created by different etiological factors, such as tumors, infections, and trauma. They can usually be treated with bone grafting procedures. For these situations, the autogenous bone graft is considered the gold standard [1, 2] because it has osteogenic potential [3]. However, the removal of autologous graft often presents a significant risk of postoperatively complications and morbidity [4]. 

A large number of bone substitute materials such as homologous, xenogeneic, and synthetic grafts are available, but also have drawbacks related to mechanical and biological properties [5]. 

 Tissue engineering has advanced recently in an attempt to reproduce lost tissues and organs, including bone tissue. Thus, several studies have been directed to the creation of cellular therapies protocols [6–8] in order to restore the native tissue without requiring the harvest of large autologous bone grafts.

The use of stem cells has been extensively related. Its ability to differentiate into a variety of specialized cells (producing adipose tissue, bone, cartilage, and endothelium) becomes the object of great interest in the tissue engineering field. Many studies have been reported in the literature using mesenchymal cells from bone marrow to maximize the results of bone repair [9–13]. This therapy promotes the use of a vital bone graft, with osteogenic potential, without the need of harvesting an autologous bone graft.

In order to increase the number of available osteoprogenitor cells, mesenchymal stem cells can undergo a series of processes, such as culture [14, 15], concentration [16], and/or incorporation into scaffolds [17, 18] before being deployed to the sites to be repaired. However, the role of mesenchymal stem cells is not limited to increasing the number of osteogenic cells. These cells also exhibit angiogenic effects *in vivo* and secrete sufficient amounts of vascular endothelial growth factor to promote angiogenesis when transplanted transplanted [19].

Despite the possible use of such processes to increase the available mesenchymal stem cells, Pelegrine et al. (2010) [12] have demonstrated good clinical results in bone repair in humans when using the autologous bone marrow without processing it. However, bone healing after the use of the autologous bone marrow incorporated into Bio-Oss, like a scaffold, was never been studied.

Therefore, the aim of this study was to evaluate the percentage of new bone formation, connective tissue, and residual graft particles with bone marrow therapy associated with Bio-Oss, with or without Bio-Gide, in inlay grafts.

## 2. Materials and Methods

### 2.1. Materials

**Figure 1:**
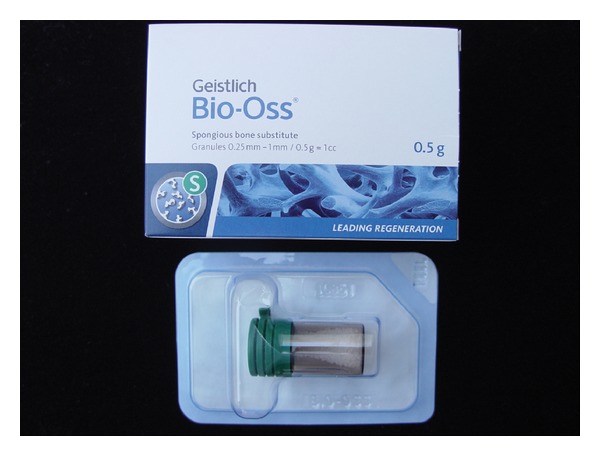
Xenograft (bovine origin) with small granules used in the study.

Bio-Gide (Geistlich Biomaterials, Wolhusen, Switzerland) is a resorbable bilaminar collagen membrane of porcine origin (Figure 1) used to prevent unwanted invasion of adjacent tissues to the bone defect, thus optimizing bone healing [20]. 

**Figure 2:**
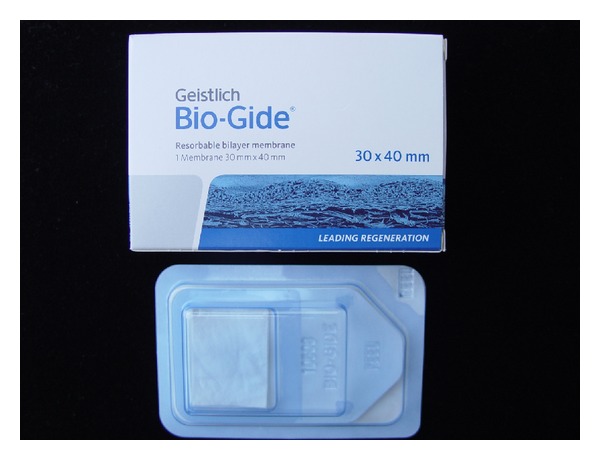
Xenogenous (porcine origin) barrier membrane used in the study.

Bio-Oss (Geistlich Biomaterials, Wolhusen, Switzerland) is a particulated xenograft bone substitute [21]. In the present study, it was used as small particles, with granules ranging from 0.25 mm to 1 mm (Figure 2).

### 2.2. Experimental Design

**Figure 3:**
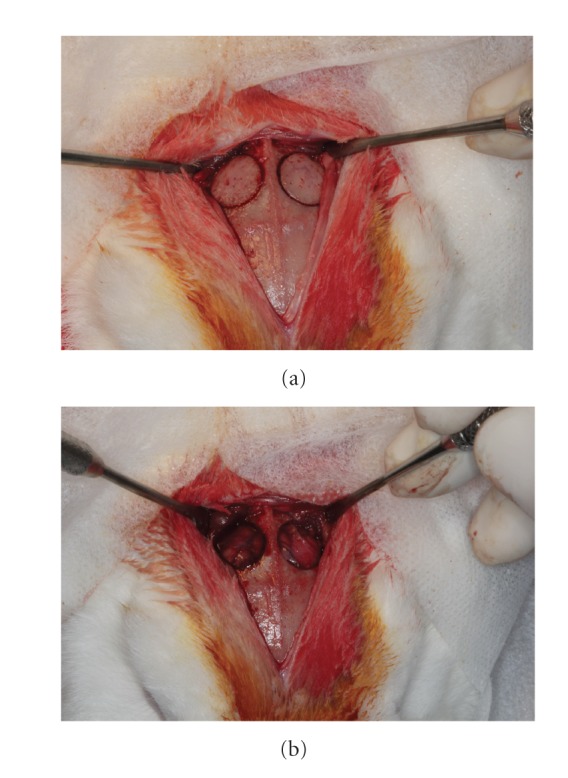
Critical-size defects in the parietal bones made with a trephine bur. (a) Before removal and (b) after removal.

Ten adult, skeletally mature New Zeland white rabbits, weighing 3.5-4 kg from the animal colony of UNIFESP (Cedeme, São Paulo, Brazil), were used in this study. The animals underwent a period of adaptation to environmental conditions prior to being housed on the premises of UNIFESP. Rooms with controlled temperature 18 to 20°C and specific individual cages for rabbits were used. The animals received food based on commercial feed pellets and water *ad libitum*. Bilateral 12 mm diameter critical-size defects were created in the parietal bones of all animals (Figures 3(a) and 3(b)).

**Figure 4:**
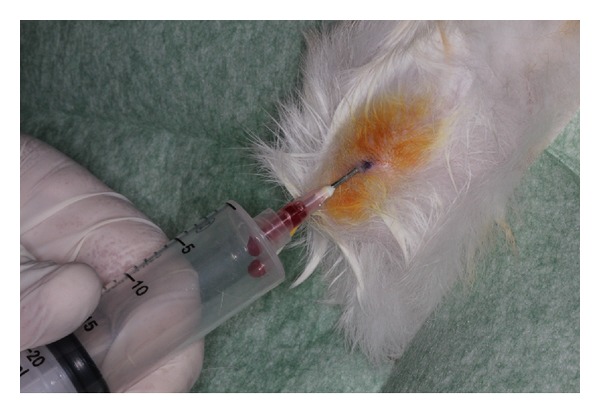
Autologous bone marrow being aspirated from the shinbone of one rabbit.

The 10 rabbits were randomly divided into two groups, where in control group animals, the bilateral calvarial defects were filled with Bio-Oss and in experimental group, the calvarial defects were filled with Bio-Oss enriched with an autologous bone marrow aspirate (Figure 4).

**Figure 5:**
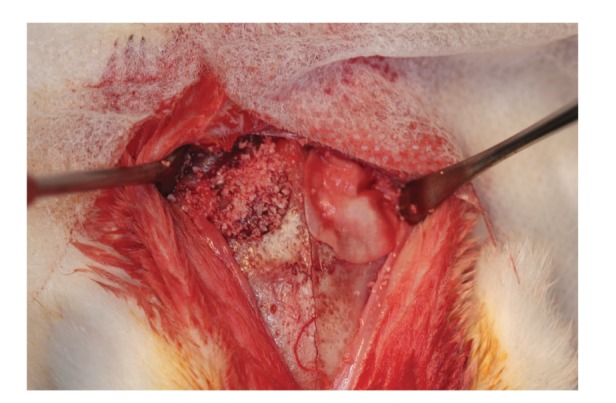
One of the parietal critical-size defects being protected with Bio-Gide membrane.

In each of the 10 animals, one of calvarial defects was randomly protected with Bio-Gide (Figure 5).

The random process was done by the program available at http://www.randomization.com/.

### 2.3. Experimental Steps

#### 2.3.1. Autologous Bone Marrow Aspiration

Under general anesthesia, bone marrow aspirates were obtained from the shinbone of the 10 rabbits using a disposable 40 × 10 needle (1.10 mm × 38 mm) with 20 mL disposable syringes embedded with heparin to prevent blood clotting (Figure 2). In the 5 experimental rabbits, the autologous bone marrow was mixed to the xenograft and in the 5 control rabbits, the autologous bone marrow was discarded. 

#### 2.3.2. Surgical Protocol

The surgical protocol for this study was analyzed and approved by the Unifesp Ethics Committee (number 2139/2011).

Anesthesia was induced by ketamine (40 mg·kg^−1^), midazolam (2 mg·kg^−1^), and fentanyl citrate (0.8 microg·kg^−1^) and maintained using a mixture of 1 : 1.5% isoflurane/N_2_O: oxygen (2/3 : 1 : 3), using a pediatric-size laryngeal mask airway. The tops of the 10 animals' heads were shaved, and the site was disinfected with a povidone-iodine solution. After administration of a local anesthetic injection of 2% lidocaine with epinephrine 1 : 100,000, a sagittal incision was made and the skin and periosteum were drawn back.

At this time, the two defects were created, one on each side of the midline, with a 12 mm diameter trephine bur (Figure 1). The grafts, with or without autologous bone marrow cells aspirate (experimental or control group, resp.), were placed directly onto the dura, replacing the volume of bone that was removed. Then, Bio-Gide membrane was randomly placed over the right or left side in each animal (Figure 5).

In both groups, the incision was closed in layers. Postoperatively, the animals received food and water *ad libitum* for the remainder of the experimental period.

**Figure 6:**
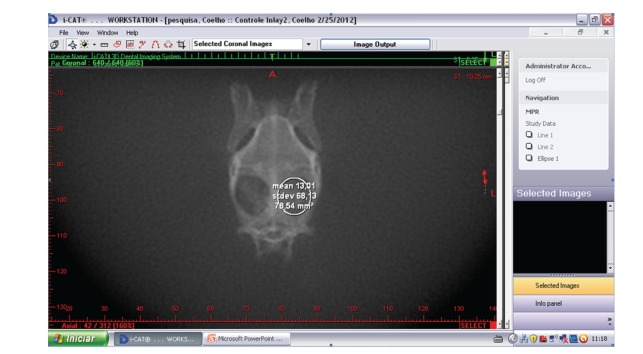
CT view after the measurement of a residual defect area.

All 10 animals were sacrified 8 weeks after the initial surgery. Their heads were removed, fixed in 10% buffered formalin, and scanned with computerized tomography (CT) (Figure 6).

**Figure 7:**
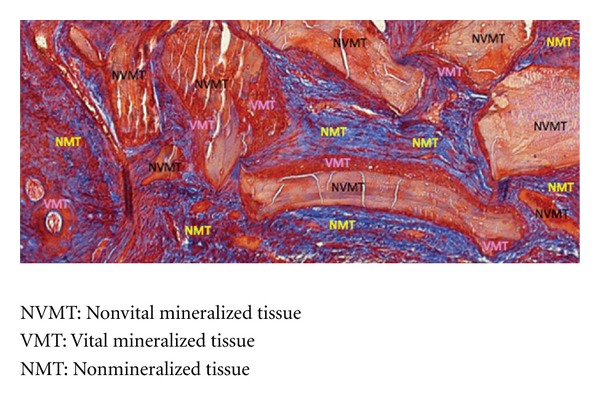
Histological view of NVMT, VMT, and NMT in 100x magnification (Mallory trichrome).

Then, their parietal bones were harvested and decalcified for histologic processing for the histomorphometrics (Figure 7). 

### 2.4. Auxiliary Services

#### 2.4.1. Computerized Tomography (CT) Analysis

All CT scans were performed with a Classic i-Cat scanner (Imaging Science International, Haltherfield, EUA). Voxel with 0.25 mm, 8.00 cm of field view, and 40 s as an exposure time were selected for all acquisition. The X-rays setting was established by equipment in 120 kV and 5 to 7 mA in accordance with the resolution. The water was not utilized to simulate a soft tissue because the rabbit's cranium was soaked in 10% buffered formalin inside a plastic recipient. The rabbit's cranium was positioned in natural way and hugged by wax. A wood support was used to keep the acquisition established.

All the images were processed in Xoran (Xoran Technologies, EUA) software on its own equipment workstation, where any slice anatomic planes correction and bone regeneration analysis were done. Besides the Angio-Sharpen-Medium 5 × 5 filter application in all the images, the contrast, and brightness were also adjusted, in order to give a better image detail for the observer.

The residual defect area, in each side of the rabbit's cranium, was measured in mm^2^ (Figure 6).

#### 2.4.2. Histologic Preparation and Histomorphometric Analysis

All specimes were decalcified by submersion in 10% EDTA for 8–12 weeks at room temperature. Each calvaria was further sectioned into an anterior and posterior portion. Both portions were embedded in paraffin blocks. Then 7 *μ*m sections of each calvarium were obtained progressively from the middle of the defects, stained with Mallory trichrome, and were qualitatively examined under light microscopy.

Digital images were captured using a CCD digital camera (RT Color; Diagnostic Instruments, Sterling Heights, MI) attached to the light microscope (Magnification X 1.25). To create a single image for each histologic section, the digital images were merged using Adobe Photoshop Elements 2.0 (Adobe Systems, San Jose, CA).

A blinded investigator has traced all the images for new bone formation using Image Pro Plus 4.5 Software for Windows (Media Cybernetics, San Diego, CA). The following parameters were measured: (1) non-vital mineralized tissue (NVMT); (2) vital mineralized tissue (VMT); and (3) non-mineralized tissue (NMT) (Figure 7).

All results were expressed as a percentage of total area of the defect. Four sections taken posterior and 4 sections taken anterior of the center of the defect were analyzed.

#### 2.4.3. Statistical Analysis

All quantitative data were analyzed with GraphPad Prism (GraphPad Software Inc., San Diego, USA). ANOVA test were used for comparisons between groups, and a  *P*  value less than 0.05 indicated statistical significance.

## 3. Results

### 3.1. Tomographic Results

**Figure 8:**
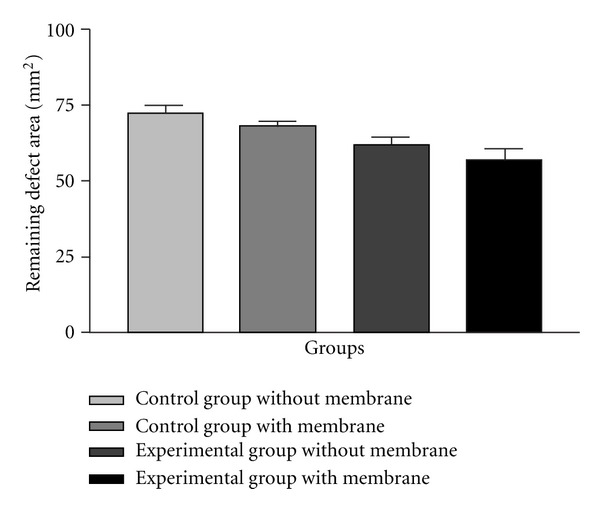
Tomographic analysis of the remaining bone defect area (in mm^2^).

The CTs analysis showed that the experimental group where the Bio-Gide was used had a statistically significant lower remaining defect area when compared with control group (both with and without Bio-Gide coverage). On the other hand, when comparing both experimental groups (with and without Bio-Gide) and both control groups (with and without Bio-Gide), it was observed no statistically significant difference (Figure 8 and Table 1).

### 3.2. Histomorphometric Results

It was verified statistically significant more new bone formation, with vital mineralized tissue (VMT), in the experimental group where the Bio-Gide was used when compared with the other groups (control groups with and without Bio-Gide and experimental group without Bio-Gide). The VMT of the control group with Bio-Gide was similar to the experimental group without Bio-Gide and, both, in a higher level than the control group without Bio-Gide. 

The non-vital mineralized tissue (NVMT) had no statistical difference between all groups.

**Figure 9:**
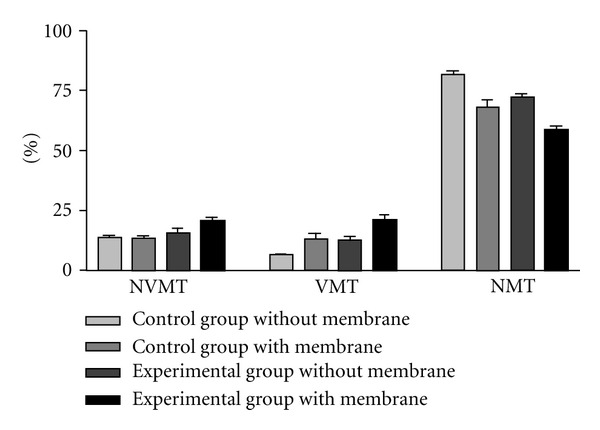
Histomorphometric analysis of the non-vital mineralized tissue (NVMT), vital mineralized tissue (VMT), and non-mineralized tissue (NMT) in percentage (%).

The non-mineralized tissue (NMT) was less prominent in the experimental group where the Bio-Gide was used when compared with the other groups (control groups with and without Bio-Gide and experimental group without Bio-Gide). The NMT of the control group with Bio-Gide was similar to the experimental group without Bio-Gide and, both, in a lower level than the control group without Bio-Gide (Table 2 and Figure 9).

## 4. Discussion

In the present study, critical size defects were created in rabbit calvaria. Critical size defects are those bone defects that cannot completely heal by itself, which was evaluated by Borie et al. (2011) [22] that stated that, in rabbit calvaria, a 8 mm diameter defect could not regenerate without the use of a bone graft. Despite this affirmation, in the present study, we have used defects of 12 mm, which allowed the attainment of a large defect that could justify the use of a new concept, like the proposed by the incorporation of an autologous bone marrow graft to a xenograft. 

The osseoconductor xenograft Bio-Oss was used in both, experimental and control groups. This material is identical to human bone, both from a chemical and physical point of view and can be used to substitute the autologous bone graft in many cases [23–26]. Recently, Sollazzo et al. (2010) [27] reported that Bio-Oss acts in the early differentiation stages of human mesenchymal cells, which could contribute to bone formation. Therefore, the use of Bio-Oss like a scaffold in the tissue engineering field seems to be reasonable.

In the control group, where the Bio-Oss was not associated with autologous bone marrow, both the tomographic and histomorphometric analysis demonstrated a lower bone level when compared to experimental group, where Bio-Oss was associated with autologous bone marrow. These better results achieved in the present study by the use of the autologous bone marrow in inlays defects corroborate Lucarelli et al. (2004) [28], Paley et al. (1986) [29], and Pelegrine et al. (2010) [12]. More recently, tomographic and histomorphometric analysis showed benefits with the adjunct use of autologous bone marrow with homologous bone graft in onlay bone reconstruction in humans, allowing dental implant installation after 6 months [30]. This might be explained by the presence of stem cells in the grafted bone marrow in the experimental group, but there are other factors in bone marrow that could contribute to these better results, such as the presence of other cells and growth factors in bone marrow.

In the present study, the bone marrow was harvested in both groups (control and experimental), despite the effective use of it just in the experimental group. It was done to standardize the rabbits' stress level between groups.

The use of a barrier membrane demonstrated better results. In the experimental group, where Bio-Gide was used, the tomographic analysis showed the lowest level of residual bone defect area. Newly formed bone progressed in a centripetal fashion reducing, consecutively, the partially mineralized central area. By the histomorphometric analysis, it was showed that the use of a barrier membrane, that can exclude the soft tissues in the early bone healing stages like Bio-Gide, increases the amount of vital mineralized tissue (VMT) and decreases the amount of non-mineralized tissue (NMT), both in control and experimental groups, corroborating Busenlechner et al. (2005) [31]. 

The NVMT represents Bio-Oss residual particles. NVMT occurred in a similar pattern in all groups, showing that, despite the better bone formation with the use of bone marrow and Bio-Gide, the resorption level of the xenograft particles was not altered. This can be considered a benefit in many procedures where the graft resorption rate needs to be slow.

The higher level of vital mineralized tissue (VMT) and the lower level of non-mineralized tissue (NMT) that were obtained with the use of Bio-Gide and autologous bone marrow graft were optimized when both were used together, suggesting a possible synergic effect between them. Therefore, the use of autologous bone marrow seems to have a similar potential of the autogenous bone graft when associated to a bone substitute material, since the inclusion of an appropriate amount of autogenous bone in the grafting material has been reported to substantially shorten the healing time when compared to sites grafted with bone substitutes alone [32].

In rabbits, the bone metabolism is approximately three times faster than in humans [33]. This fact justifies the sacrifice of the animals after eight weeks in the present study since bone augmentation procedures are usually performed six months, or 24 weeks, prior to implant placement.

The higher levels of VMT, the lower levels of NMT, and the lower remaining defect area, after 8 weeks, in the sites grafted with the autologous bone marrow associated to the guided bone regeneration technique suggest that the bone healing process is accelerated by this technique and, maybe, the quality of the healing process is maximized. It could allow more accurate bone regeneration and also the placement of a titanium implant after a lower healing time than that used today. However, this hypothesis must be confirmed by further studies with a longer healing period.

Until now, there was no published study analyzing the effectiveness of the association between Bio-Oss, Bio-Gide, and autologous bone marrow graft. Although the present study suggests that the use of autologous bone marrow and Bio-Gide, associated with Bio-Oss, presents higher levels of bone gain after 8 weeks, the potential of other methods of using stem cells such as the use of cultivated bone marrow stromal stem cells and the use of the bone marrow mononuclear fraction still needs to be compared to the presented method in animal models. 

## 5. Conclusion

Both autologous bone marrow and the barrier membrane can contribute to the enhancement of bone healing, in a quantitative and qualitative manner, and seem to have a synergic effect when used together.

## Figures and Tables

**Table 1:** Statistical comparisons between groups were significant. *P* values < 0.05 were marked (*).

Groups	*P* value
Control group without membrane versus control group with membrane	0.17
Control group without membrane versus experimental group without membrane	0.15
Control group without membrane versus experimental group with membrane	0.03*
Control group with membrane versus experimental group without membrane	0.11
Control group with membrane versus experimental group with membrane	0.04*
Experimental group without membrane versus experimental group with membrane	0.04*

**Table 2:** Mean and standard deviation of the non-vital mineralized tissue (NVMT), vital mineralized tissue (VMT), and non-mineralized tissue (NMT), analyzed by histomorphometrics, in percentage (%). *P* values < 0.05 were marked (*).

	Control group without membrane		Control group with membrane		Experimental group without membrane		Experimental group with membrane		*n*	*P* value

	Mean	SD	Mean	SD	Mean	SD	Mean	SD		
NVMT	13.64	2.34	13.35	3.13	15.31	5.22	20.29	4.17	5	0.21
VMT	6.31	1.29	12.78	5.9	12.44	7.08	21.16	3.76	5	0.04*
NMT	81.41	3.25	67.95	7.03	72.16	3.27	58.54	3.58	5	0.01*
